# Procedural and Hemodynamic Outcomes of Contemporary Self-Expanding Valves for Transcatheter Aortic Valve Replacement: A Multicenter Comparison From the AMTRAC Registry

**DOI:** 10.1016/j.shj.2026.101114

**Published:** 2026-08-11

**Authors:** Amos Levi, Yeela Talmor-Barkan, Marco Barbanti, Sofia Sammartino, Giuliano Costa, Carmelo Grasso, Ole De Backer, Davorka Lulic, Fernanda Rodriguez, Carmen Olmos, Giulia Masiero, Marianna Adamo, Dabit Arzamendi, Mario García-Gómez, Jose A. Baz, Klemen Steblovnik, Victor Mauri, Matti Adam, Hendrik Wienemann, Manuel Hein, Philipp Ruile, Bing Wei Thaddeus Soh, Kush P. Patel, Luca Branca, Rodrigo Estévez-Loureiro, Ignacio J. Amat-Santos, Darren Mylotte, Matjaz Bunc, Giuseppe Tarantini, Michael J. Mullen, Luis Nombela-Franco, Pablo Codner, Aviad Rotholz, Tsahi T. Lerman, Leor Perl, Ran Kornowski, Guy Witberg

**Affiliations:** a Department of Cardiology, Rabin Medical Centre, Petach-Tikva, Israel; b Gray Faculty of Medical and Health Sciences, Tel-Aviv University, Tel-Aviv, Israel; c The Department of Medicine and Surgery, Università degli Studi di Enna Kore, Enna, Italy; d Division of Cardiology, University of Catania, Catania, Italy; e The Heart Center, Rigshospitalet, Copenhagen University Hospital, Copenhagen, Denmark; f Instituto Cardiovascular, Hospital Clínico San Carlos, Instituto de Investigación Sanitaria del Hospital Clínico San Carlos (IdISSC), Madrid, Spain; g Department of Cardiac, Thoracic and Vascular Sciences, University of Padua Medical School, Padua, Italy; h Cardiovascular Department, Spedali Civili, Brescia, Italy; i El Servicio de Cardiología, Hospital de Sant Creu i Sant Pau Barcelona, Barcelona, Spain; j Servicio de Cardiología, CIBERCV, Hospital Clínico Universitario de Valladolid, Valladolid, Spain; k Servicio de Cardiología, Hospital Álvaro Cunqueiro, Vigo, Pontevedra, Spain; l Department of Cardiology, University Medical Centre, Ljubljana, Slovenia; m Clinic III for Internal Medicine, Faculty of Medicine and University Hospital Cologne, University of Cologne, Cologne, Germany; n Department of Cardiology and Angiology, University Heart Center Freiburg Bad Krozingen, Faculty of Medicine, University of Freiburg, Freiburg, Germany; o Department of Cardiology, Galway University Hospital, and University of Galway, Galway, Ireland; p Barts Heart Centre, St Bartholomew's Hospital, London, UK

**Keywords:** Hemodynamics, Multilevel mixed-effects models, Propensity score matching, Self-expanding valves, Transcatheter aortic valve replacement

## Abstract

**Background:**

Comparative data among contemporary self-expanding transcatheter heart valve designs remain limited. We compared clinical and hemodynamic outcomes following transcatheter aortic valve replacement using 2 leading contemporary platforms.

**Methods:**

This multicenter cohort study utilized the AMTRAC registry to evaluate consecutive patients treated with either Evolut PRO/PRO+/FX or Navitor. The primary analysis employed a 1:1 propensity score-matched cohort, utilizing multilevel mixed-effects models to adjust for center-level clustering. The primary endpoint was 1-year all-cause mortality.

**Results:**

Of 2673 patients, propensity score matching yielded a balanced cohort of 1200 patients. At 1-year, all-cause mortality was similar between groups (Navitor: 7.1% vs. Evolut: 9.2%; hazard ratio 0.75; 95% CI: 0.47–1.21; *p* = 0.24). In-hospital mortality (0.8%), periprocedural stroke (2.3%), permanent pacemaker implantation (Navitor: 14.4% vs. Evolut: 15.9%; *p* = 0.554), and paravalvular leak (*p* = 0.221) were comparable. Initial differences showing lower vascular and bleeding events with Navitor were completely attenuated after accounting for center-level clustering (vascular: *p* = 0.838; bleeding: *p* = 0.745). Conversely, postprocedural mean transaortic gradient was lower with Evolut (7.3 ± 3.9 mmHg vs. 8.6 ± 4.1 mmHg; *p* < 0.001), a hemodynamic advantage that remained highly robust after center adjustment (odds ratio 2.43; 95% CI: 1.66–3.58; *p* < 0.001). Sensitivity analyses yielded consistent results.

**Conclusions:**

Contemporary self-expanding platforms demonstrate comparable 1-year mortality and stroke rates. Periprocedural vascular and bleeding safety profiles are strongly modulated by center-specific clustering and practice variations. Conversely, the superior hemodynamic performance of the Evolut platform remains independent of institutional factors. These findings support an anatomy-driven approach to valve selection integrating patient profiles with institutional practices.

## Highlights

• AMTRAC registry patients were matched 1:1 to compare contemporary self-expanding valves.
• Primary mixed-effects models accounted for institutional clustering across centers.
• One-year mortality and in hospital stroke and pacemaker rates were similar between Navitor and Evolut platforms.
• Vascular and bleeding differences disappeared after center-level clustering adjustment.
• The Evolut platform demonstrated significantly lower postprocedural mean gradients.

## Graphical abstract

**Figure:**
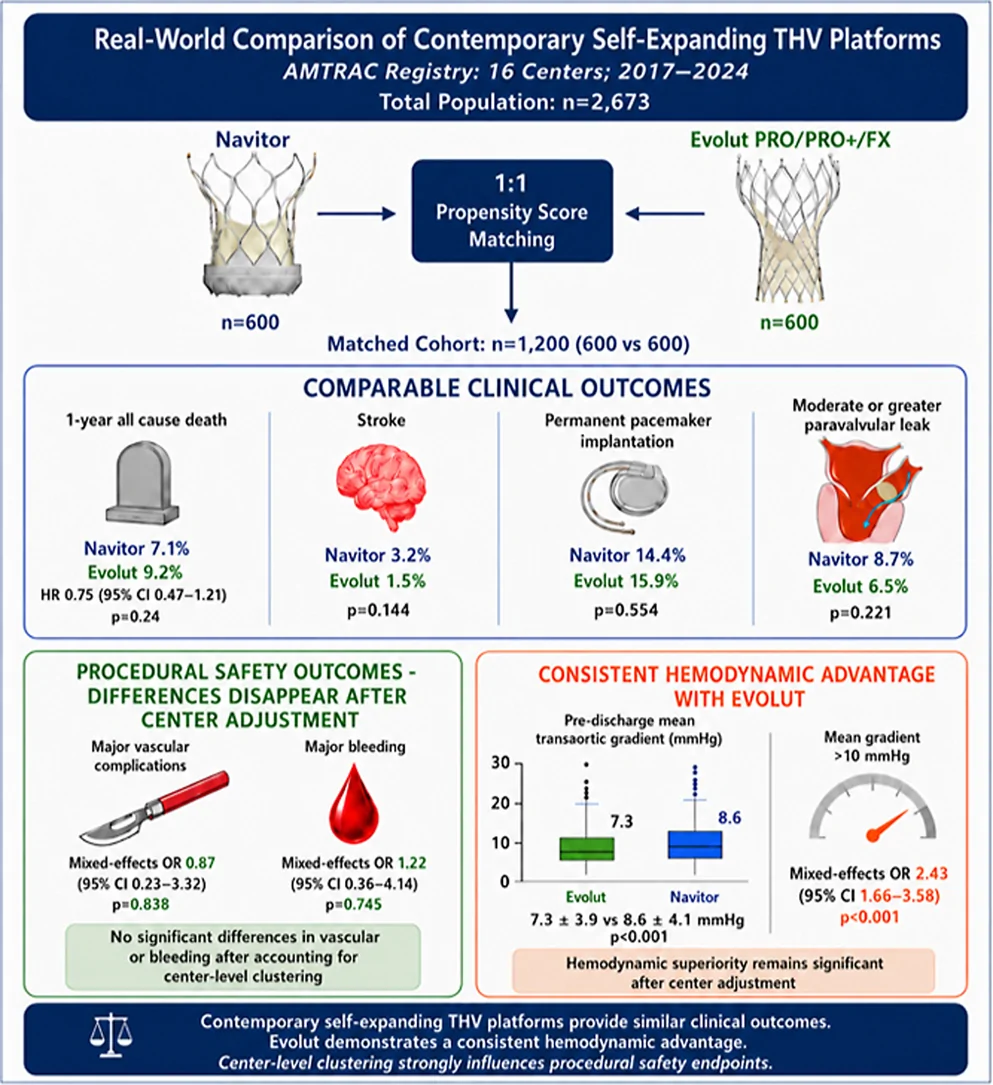

## Introduction

Transcatheter aortic valve replacement (TAVR) has emerged as the preferred treatment for severe aortic stenosis in a broad spectrum of patients.^1^ Transcatheter heart valves (THVs) can be broadly categorized into balloon-expandable valves (BEVs) and self-expanding valves (SEVs). Although BEVs tend to share similar design characteristics and deployment strategies, SEVs exhibit substantial heterogeneity in terms of frame architecture, leaflet positioning, deliverability, and hemodynamic performance. Among the SEV options currently in widespread clinical use, the 2 predominant THV types are distinguished by their leaflet position: tall frame supra-annular (Evolut; Medtronic, Minneapolis, Minnesota), and tall frame intra-annular (Navitor; Abbott, Santa Clara, California) designs.

Although comparative studies between BEV and SEV platforms exist, randomized data comparing different SEVs designs remain sparse. This represents a significant gap in the literature, particularly as device selection becomes increasingly tailored to individual patient anatomy and clinical characteristics. Existing real-world evidence often aggregates all SEVs into a single category, failing to account for the important functional and procedural distinctions among the different SEV configurations.

This study aims to provide a head-to-head comparison of clinical and echocardiographic outcomes associated with the 2 leading SEV types—Navitor and Evolut—using real-world data from a large, international registry.

## Material and Methods

### Study Design and Population

This was a retrospective observational study using data from the AMTRAC (Aortic + Mitral TRAnsCatheter) registry (ClinicalTrials.gov Identifier: NCT04031274), an investigator initiated, international, multicenter registry including over 23,000 TAVR procedures from 17 centers in Europe and Israel. The registry collects detailed baseline, procedural, and follow-up data. Local institutional review board approval was obtained at all participating centers, and data use complied with applicable ethical standards.

We included consecutive patients who underwent transfemoral TAVR with a SEV THV between registry initiation and December 31, 2024. Only procedures performed with contemporary SEV platforms were included: Evolut PRO/PRO+/FX (Medtronic), or Navitor (Abbott). Valve-in-valve procedures were excluded.

### Outcomes

The primary endpoint was all-cause 1-year mortality. Secondary endpoints included in-hospital adverse events defined by Valve Academic Research Consortium-3^2^ criteria including: permanent pacemaker implantation (PPI), stroke, major vascular complications and moderate to severe bleeding complications (Bleeding Academic Research Consortium [BARC] 3-5), and predischarge valve hemodynamics including mean transaortic gradient (mAVG) and paravalvular leak (PVL).

### Statistical Analysis

Continuous variables are presented as mean ± SD or median (interquartile range), as appropriate, and were compared using Student's t-test or the Mann–Whitney U test. Categorical variables are presented as counts and percentages and were compared using the χ^2^ test or Fisher exact test.

To reduce treatment-selection bias, patients undergoing TAVR with Navitor or Evolut valves were matched using 1:1 propensity score matching (PSM) without replacement. The propensity score was derived using a logistic regression model incorporating baseline demographic, clinical, echocardiographic, anatomical, and procedural variables including age, sex, Society of Thoracic Surgeons score, left ventricular ejection fraction, estimated glomerular filtration rate, annular dimensions, procedure year, and other clinically relevant covariates. Covariate balance was assessed using standardized mean differences, with values < 0.10 considered acceptable. Love plots were used for visualization.

Because valve selection and procedural practices may vary across participating centers, all comparative outcome analyses in the matched cohort were performed using multilevel mixed-effects models with treatment center included as a random intercept. Binary outcomes were analyzed using mixed-effects logistic regression, while time-to-event outcomes were evaluated using mixed-effects Cox proportional hazards models. The degree of between-center clustering was quantified using the intraclass correlation coefficient.

These models were considered the primary inferential analyses of the study.

Several sensitivity analyses were performed, including restriction to the contemporary era (2021–2024), exclusion of patients treated with the earlier-generation Evolut PRO valves, and exclusion of bicuspid anatomy.

Treatment effects are reported as odds ratios (ORs) with 95% Wald CIs. A 2-sided *p*-value of less than 0.05 was considered statistically significant. All analyses were performed using R (version 4.3; R Foundation for Statistical Computing, Vienna, Austria).

## Results

In total, 2673 patients who underwent TAVR with the Evolut (n = 2073) or Navitor (n = 600) platforms in 16 centers were included in the current study. Baseline characteristics of the entire cohort are described in Table 1. The distribution of TAVR valves across the centers is described in the supplementary material (Supplementary Table S1).

**Table 1:** Baseline characteristics - unadjusted analysis

	Overall	Evolut	Navitor	*p*
n	2673	2073	600	
Age, y	81.6 ± 6.4	81.9 ± 6.3	80.5 ± 6.6	<0.001
Male gender	1435 (53.7)	1148 (55.4)	287 (47.8)	0.001
LV dysfunction	305 (11.4)	235 (11.3)	70 (11.7)	0.880
COPD	397 (14.9)	273 (13.2)	124 (20.7)	<0.001
DM	840 (31.4)	670 (32.3)	170 (28.3)	0.071
Atrial fibrillation	720 (26.9)	546 (26.3)	174 (29.0)	0.214
CKD stage 4/5	222 (8.3)	183 (8.8)	39 (6.5)	0.083
STS score	4.50 ± 4.08	4.80 ± 4.05	3.50 ± 4.00	<0.001
Previous PCI	553 (20.7)	422 (20.4)	131 (21.8)	0.466
Previous CABG	177 (7.0)	142 (7.4)	35 (5.8)	0.225
Baseline mean AVG, mmHg	48 ± 16	49 ± 17	48 ± 14	0.413
Baseline pacemaker	320 (12.0)	255 (12.3)	65 (10.8)	0.366
Small annulus	1684 (63.0)	1401 (67.6)	283 (47.2)	<0.001
Bicuspid AV	114 (4.3)	98 (4.7)	16 (2.7)	0.037
Procedure y				
2017	10 (0.4)	10 (0.5)	0 (0.0)	
2018	173 (6.5)	173 (8.3)	0 (0.0)	
2019	408 (15.3)	408 (19.7)	0 (0.0)	
2020	329 (12.3)	329 (15.9)	0 (0.0)	
2021	329 (12.3)	258 (12.4)	71 (11.8)	
2022	680 (25.4)	392 (18.9)	288 (48.0)	
2023	623 (23.3)	398 (19.2)	225 (37.5)	
2024	121 (4.5)	105 (5.1)	16 (2.7)	

Data presented as mean ± SD or n (%).

Abbreviations: AV, aortic valve; AVG, aortic valve gradient; CABG, coronary artery bypass graft surgery; CKD, chronic kidney disease; COPD, chronic obstructive pulmonary disease; DM, diabetes mellitus; LV, left ventricular; PCI, percutaneous coronary intervention; STS, Society of Thoracic Surgeons.

PSM analysis yielded a balanced cohort of 1200 patients distributed equally between platforms. Love plots describing standard mean differences demonstrated appropriate matching (Supplementary Figure S1). Baseline characteristics of the matched cohort are presented in Table 2. The mean age was 80.5 ± 6.5 and females comprised 51.2% of patients. The mean Society of Thoracic Surgeons score was 3.6 ± 3.3 and mAVG was 48 ± 16.

**Table 2:** Baseline characteristics – PSM cohort

	Overall	Evolut	Navitor	*p*
n	1200	600	600	
Age, y	80.52 ± 6.54	80.56 ± 6.45	80.49 ± 6.63	0.850
Male gender	586 (48.8)	299 (49.8)	287 (47.8)	0.525
LV dysfunction	139 (11.6)	69 (11.5)	70 (11.7)	1.000
COPD	219 (18.3)	95 (15.8)	124 (20.7)	0.055
DM	358 (29.8)	188 (31.3)	170 (28.3)	0.283
Atrial fibrillation	358 (29.8)	184 (30.7)	174 (29.0)	0.570
Stage 4/5 CKD	79 (6.6)	40 (6.7)	39 (6.5)	1.00
STS score	3.6 ± 3.3	3.7 ± 2.5	3.5 ± 4.0	0.269
Previous PCI	243 (20.2)	112 (18.7)	131 (21.8)	0.196
Previous CABG	69 (6.0)	34 (6.2)	35 (5.8)	0.901
Base mean AVG	48 ± 16	48 ± 18	48 ± 14	0.971
Baseline pacemaker	116 (9.7)	51 (8.5)	65 (10.8)	0.204
Small annulus	583 (48.6)	300 (50.0)	283 (47.2)	0.355
Bicuspid AV	36 (3.0)	20 (3.3)	16 (2.7)	0.612
Procedure y				
2019	10 (0.8)	10 (1.7)	0 (0.0)	
2020	25 (2.1)	25 (4.2)	0 (0.0)	
2021	128 (10.7)	57 (9.5)	71 (11.8)	
2022	472 (39.3)	184 (30.7)	288 (48.0)	
2023	480 (40.0)	255 (42.5)	225 (37.5)	
2024	85 (7.1)	69 (11.5)	16 (2.7)	

Data presented as mean ± SD or n (%).

Abbreviations: AV, aortic valve; AVG, aortic valve gradient; CABG, coronary artery bypass graft surgery; CKD, chronic kidney disease; COPD, chronic obstructive pulmonary disease; DM, diabetes mellitus; LV, left ventricular; PCI, percutaneous coronary intervention; PSM, propensity score matching; STS, Society of Thoracic Surgeons.

Kaplan–Meier survival curves of up to 1 year are described in Figure 1. One-year all cause death was 9.2% and 7.1% in the Evolut and Navitor groups, respectively (HR [ref: Evolut]: 0.75; 95% CI, 0.47-1.21; *p* = 0.24). In hospital outcomes of the PSM cohort are presented in Table 3. In-hospital death occurred in 10 (0.8%) patients, without significant variability between groups. Periprocedural stroke occurred in 28 patients (2.3%), of which 4 (0.3%) were disabling; strokes were evenly distributed between the groups. PPI was reported in 162 (15.1%) of patients without a pacemaker at baseline, 14.4% and 15.9% in the Evolut and Navitor groups, respectively (*p* = 0.554). Annular rupture, tamponade, and coronary occlusion occurred infrequently and without significant variability in distribution.

**Figure 1:**
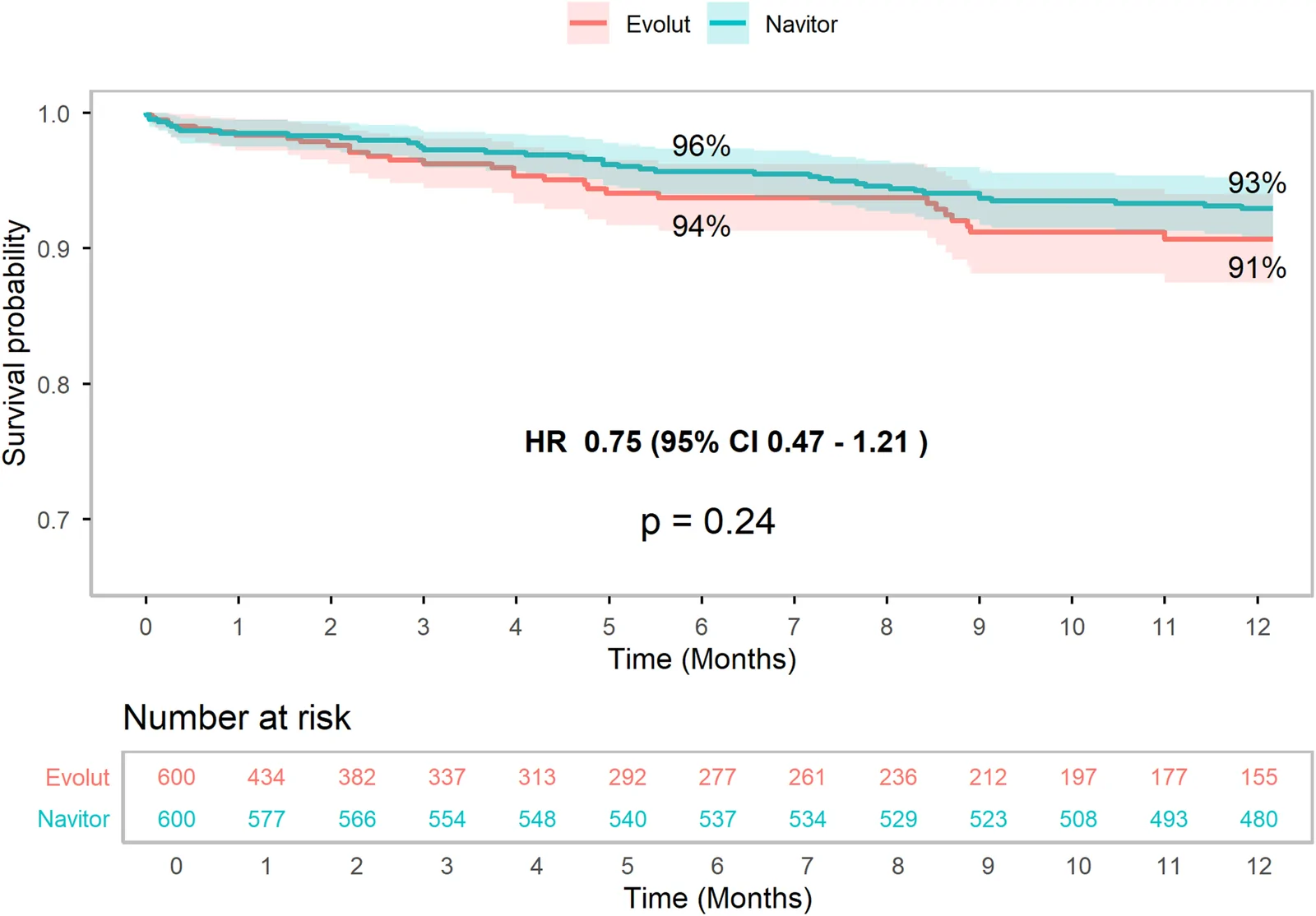
**Kaplan–Meier Curves of 1-Year Survival After TAVR With Different SEV Platforms.** Abbreviations: HR, hazard ratio; SEV, self-expanding valves; TAVR, transcatheter aortic valve replacement.

**Table 3:** Outcomes: PSM cohort and multilevel mixed-effects regression. Fixed effect: Valve (ref: Evolut). Random effect: center. n = 1200

Outcome	Evolut	Navitor	OR/β	95% CI	*p* value	ICC	AIC
Binary outcomes — logistic mixed model (OR)							
Moderate/severe Bleeding	37/600 (6.2%)	13/600 (2.2%)	1.22	0.36 – 4.14	0.745	0.147	402.8
Major vascular complication	28/600 (4.7%)	9/600 (1.5%)	0.87	0.23 – 3.32	0.838	0.237	316.8
PPI	79/549 (14.4%)	83/523 (15.9%)	1.00	0.55 – 1.81	0.989	0.156	899.0
Postprocedural PVL > mild	34/524 (6.5%)	50/577 (8.7%)	0.71	0.29 – 1.72	0.446	0.299	498.1
High postprocedural mean gradient	40/308 (13.0%)	138/518 (26.6%)	2.43	1.66 – 3.58	<0.001	0.059	844.4
In-hospital death	5/600 (0.8%)	5/600 (0.8%)	1.00	0.29 – 3.47	1.000	0.000	121.7
Stroke	9/600 (1.5%)	19/600 (3.2%)	2.18	0.77 – 6.20	0.144	0.137	265.4
Coronary occlusion	5/600 (0.8%)	4/600 (0.7%)	1.56	0.28 – 8.58	0.611	0.240	108.8
Continuous outcome — linear mixed model (β, mmHg)							
Postprocedural mAVG	7.30 ± 3.93 (n = 308)	8.58 ± 4.05 (n = 518)	+1.28β	0.72 – 1.85	<0.001	0.023	4642.2

Binary outcomes: OR, odds ratio (logistic mixed model); OR > 1, higher odds with Navitor; 95% CI, Wald CI, continuous outcome (postmean): β, mean difference in mmHg (linear mixed model, Satterthwaite df); positive β, higher gradient with Navitor, reference group for both models: Evolut. *p* < 0.05 = statistically significant.

Abbreviations: AIC, Akaike information criterion; ICC, intraclass correlation coefficient (between-center variance); mAVG, mean transaortic gradient; PPI, permanent pacemaker implantation; PSM, propensity score matching; PVL, paravalvular leak.

Major vascular complications occurred in 4.7% and 1.5% of patients in the Evolut and Navitor groups, respectively (*p* = 0.003). Moderate or severe bleeding (Valve Academic Research Consortium-3 type 2-4) occurred in 6.2% and 2.2% of patients in the Evolut and Navitor groups, respectively (*p* = 0.001). Although unadjusted analyses demonstrated lower rates of major vascular complications and moderate/severe bleeding among patients treated with Navitor, these differences were no longer significant after accounting for center-level clustering using mixed-effects regression models (major vascular complications: OR 1.00; 95% CI 0.55-1.81, *p* = 0.838; bleeding: OR 1.22; 95% CI 0.36-4.14, *p* = 0.745).

In terms of hemodynamic outcomes, post procedural mAVG declined to 7.3 ± 4 mmHg and 8.6 ± 4 mmHg in the Evolut and Navitor groups, respectively (*p* < 0.001; Figure 2, panel A). This translated to mAVG >10 mmHg in 13.0% and 26.6% in the Evolut and Navitor groups, respectively (*p* < 0.001). The likelihood of a residual mean gradient >10 mmHg remained significantly higher with Navitor after mixed-effects adjustment (OR 2.43, 95% CI 1.66–3.58; *p* < 0.001). PVL greater than mild was recorded in 6.5% and 8.7% of the patients in Evolut and Navitor groups, respectively (*p* = 0.211; Figure 2, panel B).

**Figure 2:**
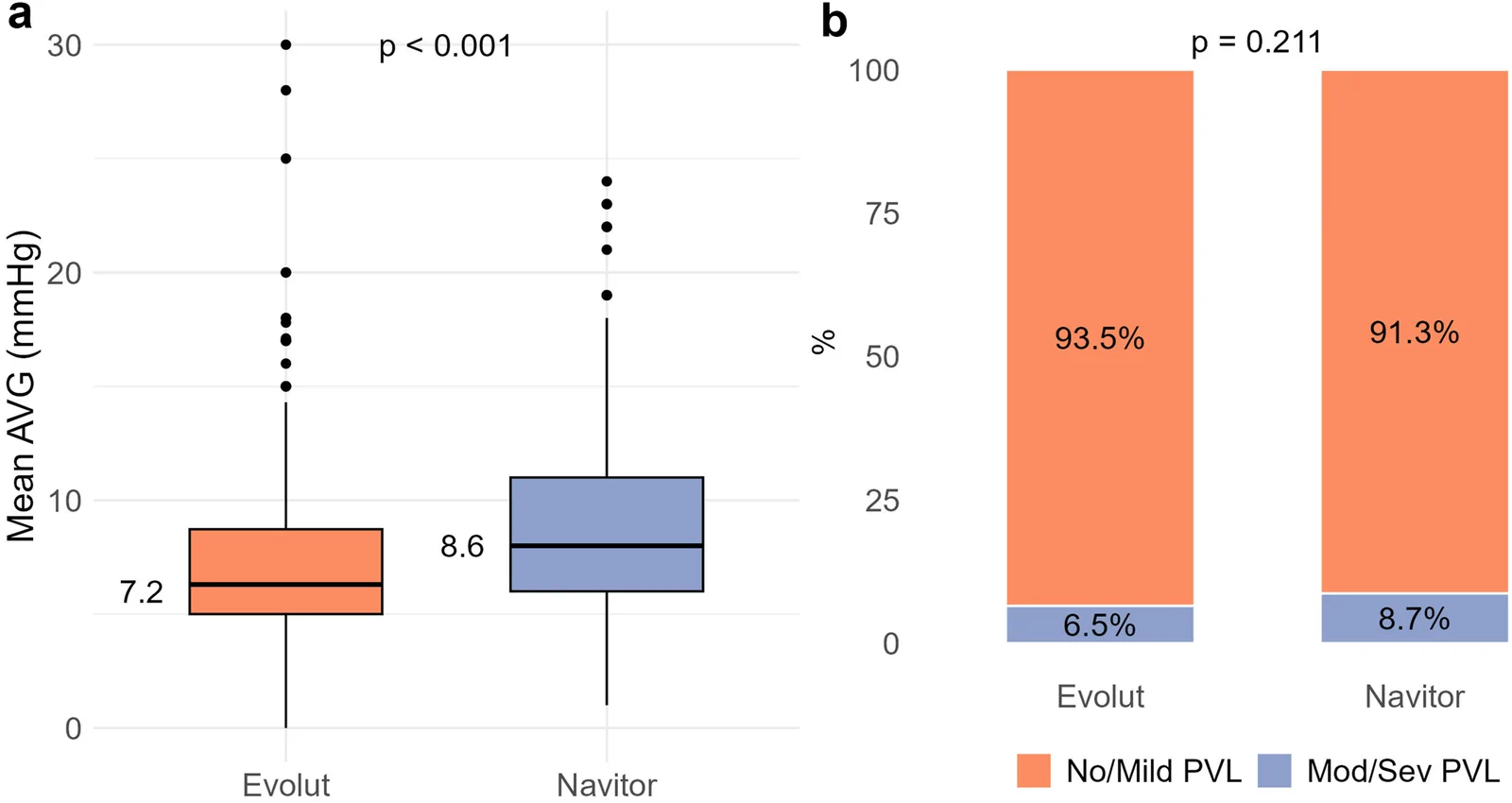
**Post Procedural Hemodynamic Outcomes.(a)** Mean post procedural transaortic gradient in the Evolut and Navitor groups; **(b)** stacked bar plot representing the rate of moderate/severe PVL in the Evolut and Navitor groups. Abbreviations: AVG, aortic valve gradient; PVL, paravalvular leak.

### Sensitivity Analyses

Restricting the analysis to the contemporary era (2021–2024, n = 1156), the exclusion of Evolut PRO from the cohort (Evolut PRO+/FX vs. Navitor, n = 904), and the exclusion of bicuspid aortic valve anatomies (n = 1130) yielded similar overall findings, with consistent hemodynamic superiority of Evolut and descriptively lower rates of vascular and bleeding complications among Navitor recipients. (Supplementary Tables S3 and S4).

## Discussion

In the current study, we compared outcomes of patients undergoing TAVR with the Evolut and Navitor SEV-THV platforms in a 1:1 PSM matched cohort. A total of 1200 patients were matched. Our main findings are as follows: (1) all-cause mortality at 1 year was similar in both groups; (2) rates of in-hospital death, permanent pacemaker implantation, and stroke were comparable between groups; (3) descriptive analyses demonstrated lower rates of major vascular and bleeding complications with Navitor; however, these differences were completely attenuated after accounting for center-level clustering; (4) Evolut showed superior valve hemodynamics with lower mAVG, but similar performance in terms of PVL; the hemodynamic advantage of Evolut remained highly robust even after random intercept adjustment (OR: 2.43, 95% CI: 1.66–3.58, *p* < 0.001); and (5) sensitivity analyses confined the cohort to the contemporary era and contemporary valve iterations were highly consistent with the primary findings.

Randomized controlled trials and large observational studies consistently show that clinical outcomes with the SAPIEN (BEV) and Evolut (SEV) THV platforms are broadly similar with regards to all-cause mortality and disabling stroke at 30 days and 1 year. However, there are important device-specific differences in periprocedural outcomes: Evolut is associated with higher rates of new PPI and moderate/severe PVL, but lower mean gradients and larger effective orifice area compared to SAPIEN.^3^, ^4^, ^5^, ^6^

These considerations collectively suggest that TAVR centers should maintain access to both BE and SE THV platforms to ensure procedural flexibility and optimize outcomes across diverse clinical/anatomical scenarios. Although the BEV landscape is largely dominated by a single platform, the SEV space comprises several systems that differ in key structural characteristics, including leaflet position (supra-annular vs. intra-annular), frame architecture (cell size, frame geometry, and frame height), and radial force profile.

This heterogeneity raises an important question: Is there a need for multiple SEV platforms, or could a single platform adequately address the spectrum of clinical and anatomical requirements? Furthermore, the structural diversity across SEV valves provides a strong rationale for comparative evaluation of their performance. Accordingly, we sought to investigate whether meaningful differences exist in clinical and hemodynamic outcomes among currently available SEV platforms.

Prior comparative data between self-expanding TAVR platforms are limited by their inclusion of older-generation or completely discontinued device iterations, rendering historical benchmarks largely irrelevant to modern clinical practice. The recently published HERA-TAVI registry^7^ represents a contemporary exception, offering the largest head-to-head comparison to date between current-generation intra-annular SEVs (Navitor/Navitor Vision) and latest-generation supra-annular SEVs (Evolut FX/FX+). Our findings stand in continuum with HERA-TAVI, which similarly demonstrated overall clinical equity and comparable 1-year survival profiles between the 2 contemporary platforms (composite primary endpoint: 12.6% vs. 11.3%, *p* = 0.422). Similarly, our primary matched cohort experienced low, and overall comparable, rates of 1-year mortality, stroke, and in-hospital death.

However, compelling nuances emerge when examining procedural and clinical safety trade-offs through advanced statistical lenses. In our primary unadjusted matched analysis, Navitor demonstrated a significantly lower risk of major vascular complications and a favorable trend in bleeding events, an outcome that aligns with the major bleeding benefits documented in the HERA-TAVI population. Yet, when we subjected our matched cohort to a multilevel mixed-effects model to account for institutional clustering, these differences were completely attenuated (vascular complications: *p* = 0.838; bleeding: *p* = 0.745). This indicates that periprocedural access safety endpoints are heavily modulated by center-specific vascular protocols, sheath management, and closure strategies rather than being purely device-inherent. It should be emphasized that granular preprocedural computerized tomography metrics—such as access vessel tortuosity, minimum calcification diameters, and specialized safety techniques (e.g., ultrasound-guided access) were not systematically captured in our registry, introducing potential residual selection bias.

Conversely, the Evolut platform demonstrated superior hemodynamic performance, reflected by lower postimplant aortic valve gradients. These findings may be explained by the supra-annular leaflet design which offers large effective orifice areas and the higher radial force profile relative to other platforms, potentially conferring an advantage in smaller annular anatomies.^6^ Regarding paravalvular regurgitation, our primary cohort demonstrated no statistically significant difference in rates of PVL greater than mild (Evolut: 6.5% vs. Navitor: 8.7%, *p* = 0.211). This lack of a divergent signal is in complete alignment with the contemporary 1-year HERA-TAVI data, where both platforms achieved similarly excellent, low rates of moderate-to-severe PVL. This reinforces that the active external sealing cuff incorporated into the updated Navitor frame successfully addresses the paravalvular regurgitation vulnerabilities seen in older-generation intra-annular iterations. Although the gradients in the Navitor group were statistically higher in our study, the absolute difference in mean post-TAVR gradient was small (1.3 mmHg) and arguably clinically meaningless. In this context, it has been recently shown that higher postprocedural mAVG in patients with small aortic annuli undergoing BEV vs. SEV implantation did not translate to inferior clinical outcomes at 5 years of follow-up.^8^

Regarding PPI, our primary cohort observed comparable rates across groups (14.4% for Evolut vs. 15.9% for Navitor, *p* = 0.554). Interestingly, this stands in notable contrast to the modern HERA-TAVI registry experience, where new PPI remained significantly more frequent following intra-annular Navitor implantation compared to the supra-annular platform (22.1% vs. 16.3%, *p* = 0.007).^7^ Conduction disturbances requiring a PPI have long been considered an “Achilles' heel” of TAVR compared to surgery and are more frequently associated with SEV.^9^ However, lower rates of PPI have been consistently achieved in contemporary practice when utilizing the left and right cusp overlap view, to achieve implantation at a shallower, optimal depth.^10^ We attribute our favorable Navitor PPI profile to institutional proficiency and the strict, routine adoption of standardized projection optimization techniques which facilitate predictably shallow implant depths and minimize direct mechanical interaction with the conduction system. Furthermore, our multilevel mixed-effects modeling demonstrated that institutional clustering accounted for nearly one-sixth of the overall pacing variance (intraclass correlation coefficient = 0.156). This substantial center effect underscores that PPI rates are heavily modulated not only by center-specific variations in deployment techniques but also by local electrophysiological practice variations. Specifically, some centers routinely employ a more liberal pacing policy for transient, periprocedural conduction disturbances (such as new-onset left bundle branch block or transient advanced atrioventricular block), whereas others strictly adhere to conservative, prolonged watchful waiting protocols before committing to a permanent device.

Finally, it should be noted that additional factors that may influence THV selection were not captured in the present analysis, including long-term durability, the risk of hypoattenuated leaflet thickening or hypoattenuation affecting motion, and the feasibility of coronary access following TAVR or future valve-in-valve TAVR procedures. These issues play an important role in the lifetime management of severe aortic stenosis, are thus particularly relevant in younger patients or those with longer life expectancy, and should be the target of future investigation.^11^

### Limitations

This study has several limitations. First, its retrospective design inherently introduces the potential for selection bias and residual confounding, despite the use of robust statistical adjustments including PSM and mixed-effects modeling. Second, the absence of a centralized core laboratory for imaging assessment may have led to interobserver variability in the evaluation of PVL and valve hemodynamics. Third, quantitative calcium scoring and baseline aortic valve and left ventricular outflow tract calcification burden was missing for a significant portion of our historic cohort and could not be included in the propensity model. Fourth, electrocardiogram data were not available in this analysis; therefore, PPI rates could not be adjusted for underlying conduction disturbances before the TAVR procedure and therefore should be interpreted with caution. Finally, follow-up was limited to 1 year, precluding evaluation of longer-term clinical outcomes, structural valve durability, and late conduction disturbances, whereas the lack of intermediate, adjudicated 30-day endpoints limits our ability to isolate early postdischarge event distributions.

## Conclusions

In this large multicenter PSM analysis, contemporary self-expanding THV platforms demonstrated similar rates of mortality, stroke, vascular complications, bleeding, pacemaker implantation, and PVL after accounting for center-level clustering. The Evolut platform consistently demonstrated lower residual transvalvular gradients, representing the principal device-related difference observed between platforms. These findings underscore the importance of an individualized, anatomy-driven approach to device selection that integrates patient-specific baseline profiles with institutional practices in contemporary TAVR practice. Although randomized comparisons among current-generation SEVs remain limited, further long-term studies are warranted to assess structural valve durability, valve-in-valve compatibility, and coronary access across platforms.

## Ethics Statement

The authors confirm that this research was carried out in accordance with the appropriate ethical guidelines for human subjects. The study protocol was approved by the local institutional review boards of all participating registry centers. Due to the retrospective, observational nature of this registry database study, the requirement for individual patient informed consent was waived by the reviewing ethics committees in accordance with institutional protocols and local legislation.

## Funding

The authors have no funding to report.

## Declaration of Generative AI and AI-Assisted Technologies in the Writing Process

Artificial intelligence tools, including ChatGPT (OpenAI), were used to assist in language editing, formatting, and improving the clarity of the manuscript. All content was reviewed and verified by the authors to ensure accuracy and integrity. No AI tools were used for data analysis, image generation, or interpretation of results.

## Disclosure Statement

Amos Levi reports receiving speaker honoraria from Abbott and Boston Scientific. Yeela Talmor-Barkan reports receiving speaker honoraria from Abbott. Marianna Adamo reports receiving speaker honoraria from Abbott and Edwards Lifesciences. Matti Adam reports receiving honoraria from Abbott, Boston Scientific, Edwards Lifesciences, JenaValve Technology, Medtronic, and Meril Life Sciences. Hendrik Wienemann reports consultancy fees and travel grants from JenaValve Technology. Rodrigo Estévez-Loureiro reports serving as consultant and proctor for Abbott Vascular, Edwards Lifesciences, Boston Scientific, Valgen, Jenscare, and Venus Medtech. Ignacio J. Amat-Santos reports receiving proctor/consultation fees from Boston Scientific, Medtronic, Meril Life, and Microport. Matjaz Bunc reports serving as proctor for Meril, Abbott, Edwards, and Medtronic.

The other authors had no conflicts to declare.

## References

[1] Lerman T.T., Greenberg N., Kheifets M. (2025). Transcatheter aortic valve implantation vs surgical aortic valve replacement in patients at lower surgical risk: meta-analysis of randomized trials. Can J Cardiol.

[2] Généreux P., Piazza N., Alu M.C. (2021). Valve academic research consortium 3: updated endpoint definitions for aortic valve clinical research. J Am Coll Cardiol.

[3] Thiele H., Kurz T., Feistritzer H.-J. (2020). Comparison of newer generation self-expandable vs. balloon-expandable valves in transcatheter aortic valve implantation: the randomized SOLVE-TAVI trial. Eur Heart J.

[4] Lerman T.T., Levi A., Kornowski R. (2023). Meta-analysis of short- and long-term clinical outcomes of the self-expanding Evolut R/pro valve versus the balloon-expandable Sapien 3 valve for transcatheter aortic valve implantation. Int J Cardiol.

[5] Deharo P., Bisson A., Herbert J. (2020). Impact of Sapien 3 balloon-expandable versus evolut R self-expandable transcatheter aortic valve implantation in patients with aortic stenosis: data from a nationwide analysis. Circulation.

[6] Herrmann H.C., Mehran R., Blackman D.J. (2024). Self-expanding or balloon-expandable TAVR in patients with a small aortic annulus. N Engl J Med.

[7] Casenghi M., De Backer O., De Biase C. (2026). Clinical and haemodynamic outcomes with contemporary intra- and supra-annular self-expanding valves: the multicentre international HERA-TAVI registry. EuroIntervention.

[8] Pawar S., Patel V., Nagasaka T. (2026). Long-term clinical outcomes of balloon-expandable versus self-expanding transcatheter aortic valve replacement in patients with small aortic annulus. JAHA.

[9] Sammour Y., Krishnaswamy A., Kumar A. (2021). Incidence, predictors, and implications of permanent pacemaker requirement after Transcatheter aortic valve replacement. JACC Cardiovasc Interventions.

[10] Sá M.P., Van den Eynde J., Jacquemyn X. (2023). Cusp-overlap versus coplanar view in transcatheter aortic valve implantation with self-expandable valves: a meta-analysis of comparative studies. Catheter Cardiovasc Interv.

[11] Russo G., Tang G.H.L., Sangiorgi G. (2022). Lifetime management of aortic stenosis: transcatheter versus surgical treatment for young and low-risk patients. Circ Cardiovasc Interv.

