# Supplementary material for: Procedural and Hemodynamic Outcomes of Contemporary Self-Expanding Valves for Transcatheter Aortic Valve Replacement: A Multicenter Comparison From the AMTRAC Registry
Source: Struct Heart. 2026 Aug 11;10(10):101114. doi: 10.1016/j.shj.2026.101114 (PMC13629322; doi:10.1016/j.shj.2026.101114)
Supplement: Supplementary file 1 [file mmc1.docx]

**Supplementary material**

**Figure 1 – Love plots describing standardized mean differences distribution for the various covariates used in the propensity score matching, in the unmatched (red) and matched (green) cohorts, for (A) the primary PSM cohort, for sensitivity analysis 1 cohort (B), sensitivity analysis 2 cohort (C); and sensitivity analysis 3 cohort (D)**

**(A)**

**(B)**

**(C)**

**(D)**

**Tables**

**Table 1 – Device distribution across centers**

| Center | **Evolut** | **Navitor** | **Overall** |
| --- | --- | --- | --- |
| **#1** | 585 | 1 | 586 |
| **#2** | 542 | 26 | 568 |
| **#3** | 0 | 404 | 404 |
| **#4** | 288 | 62 | 350 |
| **#5** | 313 | 0 | 313 |
| **#6** | 225 | 2 | 227 |
| **#7** | 157 | 0 | 157 |
| **#8** | 123 | 31 | 154 |
| **#9** | 13 | 132 | 145 |
| **#10** | 132 | 0 | 132 |
| **#11** | 58 | 0 | 58 |
| **#12** | 52 | 0 | 52 |
| **#13** | 21 | 0 | 21 |
| **#14** | 9 | 0 | 9 |
| **#15** | 0 | 4 | 4 |
| #16 | 1 | 0 | 1 |

**Table 2 – outcomes – sensitivity analysis 1 – inclusion of contemporary procedures only (2021-2024)**

|  | **Overall** | **Evolut** | **Navitor** | **P** |
| --- | --- | --- | --- | --- |
| n | 1156 | 578 | 578 |  |
| In hospital death (%) | 12 (1.0) | 7 (1.2) | 5 (0.9) | 0.772 |
| Stroke (%) |  |  |  | 0.126 |
| Non disabling stroke | 23 (2.0) | 9 (1.6) | 14 (2.4) |  |
| Disabling stroke | 3 (0.3) | 0 (0.0) | 3 (0.5) |  |
| Vascular complications (%) |  |  |  | 0.119 |
| Minor (%) | 73 (6.3) | 49 (8.5) | 24 (4.2) |  |
| Major (%) | 27 (2.3) | 18 (3.1) | 9 (1.6) |  |
| Bleeding (%) |  |  |  | 0.032 |
| Moderate | 30 (2.6) | 21 (3.6) | 9 (1.6) |  |
| Severe | 8 (0.7) | 5 (0.9) | 3 (0.5) |  |
| Acute kidney injury (%) | 7 (0.7) | 7 (1.4) | 0 (0.0) | 0.016 |
| Annular rupture (%) | 2 (0.2) | 1 (0.2) | 1 (0.2) | 1.000 |
| coronary occlusion (%) | 7 (0.6) | 3 (0.5) | 4 (0.7) | 1.000 |
| Pacemaker (%) | 153 (15.0) | 73 (14.1) | 80 (15.8) | 0.502 |
| Pre discharge mean AVG (mean (SD)) | 8.1 (4.1) | 7.2 (4.0) | 8.6 (4.1) | <0.001 |
| Mean pre discharge AVG > 10 mmHg (%) | 174 (21.8) | 41 (13.8) | 133 (26.7) | <0.001 |
| Pre discharge PVL (%) |  |  |  | 0.753 |
| Moderate | 80 (7.5) | 37 (7.3) | 43 (7.7) |  |
| Severe | 10 (0.9) | 4 (0.8) | 6 (1.1) |  |
| Abbreviations: AKI = acute kidney injury; MI = myocardial infarct; AVG = aortic valve gradient; PVL = paravalvular leak | | | | |

**Table 3 - outcomes – sensitivity analysis 2 – Exclusion of Evolut PRO platform**

|  | **Overall** | **Evolut** | **Navitor** | **P** |
| --- | --- | --- | --- | --- |
| n | 904 | 452 | 452 |  |
| In hospital death (%) | 10 (1.1) | 6 (1.3) | 4 (0.9) | 0.750 |
| Stroke (%) |  |  |  | 0.541 |
| Non disabling stroke | 5 (0.6) | 2 (0.4) | 3 (0.7) |  |
| Disabling stroke | 5 (0.6) | 2 (0.4) | 3 (0.7) |  |
| Vascular complications (%) |  |  |  | <0.001 |
| Minor (%) | 26 (2.9) | 21 (4.6) | 5 (1.1) |  |
| Major (%) | 26 (2.9) | 21 (4.6) | 5 (1.1) |  |
| Bleeding (%) |  |  |  | <0.001 |
| Moderate | 8 (0.9) | 5 (1.1) | 3 (0.7) |  |
| Severe | 8 (0.9) | 5 (1.1) | 3 (0.7) |  |
| Acute kidney injury (%) | 6 (0.7) | 6 (1.4) | 0 (0.0) | 0.042 |
| Annular rupture (%) | 1 (0.1) | 0 (0.0) | 1 (0.2) | 1.000 |
| coronary occlusion (%) | 6 (0.7) | 2 (0.4) | 4 (0.9) | 0.682 |
| Pacemaker (%) | 119 (14.6) | 53 (12.9) | 66 (16.3) | 0.202 |
| Pre discharge mean AVG (mean (SD)) | 8.1 (4.0) | 7.4 (4.0) | 8.5 (4.0) | 0.001 |
| Mean pre discharge AVG > 10 mmHg (%) | 124 (20.8) | 27 (13.0) | 97 (24.9) | 0.001 |
| Pre discharge PVL (%) |  |  |  | <0.001 |
| Moderate | 62 (7.3) | 26 (6.4) | 36 (8.2) |  |
| Severe | 5 (0.6) | 1 (0.2) | 4 (0.9) |  |
| Abbreviations: AKI = acute kidney injury; MI = myocardial infarct; AVG = aortic valve gradient; PVL = paravalvular leak | | | | |

**Table 4 - outcomes – sensitivity analysis 3 – Exclusion of Bicuspid aortic valve**

|  | **Overall** | **Evolut** | **Navitor** | **פ** |
| --- | --- | --- | --- | --- |
| n | 1130 | 565 | 565 |  |
| In hospital death (%) | 10 (0.9) | 6 (1.1) | 4 (0.7) | 0.751 |
| Stroke (%) |  |  |  | 0.160 |
| Non disabling stroke | 26 (2.3) | 11 (1.9) | 15 (2.7) |  |
| Disabling stroke | 3 (0.3) | 0 (0.0) | 3 (0.5) |  |
| Vascular complications (%) |  |  |  | <0.001 |
| Minor (%) | 73 (6.5) | 48 (8.5) | 25 (4.4) |  |
| Major (%) | 33 (2.9) | 24 (4.2) | 9 (1.6) |  |
| Bleeding (%) |  |  |  | <0.001 |
| Moderate | 32 (2.8) | 22 (3.9) | 10(1.8) |  |
| Severe | 7 (0.6) | 5 (0.9) | 2 (0.4) |  |
| Acute kidney injury (%) | 8 (0.8) | 8 (1.6) | 0 (0.0) | 0.009 |
| Annular rupture (%) | 2 (0.2) | 1 (0.2) | 1 (0.2) | 1.000 |
| coronary occlusion (%) | 6 (0.5) | 2 (0.4) | 4 (0.7) | 0.682 |
| Pacemaker (%) | 157 (15.8) | 76 (15.2) | 81 (16.4) | 0.667 |
| Pre discharge mean AVG (mean (SD)) | 8.20 (4.22) | 7.48 (4.32) | 8.62 (4.10) | <0.001 |
| Mean pre discharge AVG > 10 mmHg (%) | 180 (23.1) | 45 (15.6) | 135 (27.5) | <0.001 |
| Pre discharge PVL (%) |  |  |  | 0.764 |
| Moderate | 77 (7.3) | 39 (7.8) | 38 (7.0) |  |
| Severe | 11 (1.0) | 5 (1.0) | 6 (1.1) |  |
| Abbreviations: AKI = acute kidney injury; MI = myocardial infarct; AVG = aortic valve gradient; PVL = paravalvular leak | | | | |
